# Neurons gating behavior—developmental, molecular and functional features of neurons in the Substantia Nigra pars reticulata

**DOI:** 10.3389/fnins.2022.976209

**Published:** 2022-09-06

**Authors:** Juha Partanen, Kaia Achim

**Affiliations:** Molecular and Integrative Biosciences Research Programme, Faculty of Biological and Environmental Sciences, University of Helsinki, Helsinki, Finland

**Keywords:** Substantia Nigra pars reticulata, basal ganglia, GABAergic neuron, neurogenesis, movement, seizure, sleep, reward

## Abstract

The Substantia Nigra pars reticulata (SNpr) is the major information output site of the basal ganglia network and instrumental for the activation and adjustment of movement, regulation of the behavioral state and response to reward. Due to both overlapping and unique input and output connections, the SNpr might also have signal integration capacity and contribute to action selection. How the SNpr regulates these multiple functions remains incompletely understood. The SNpr is located in the ventral midbrain and is composed primarily of inhibitory GABAergic projection neurons that are heterogeneous in their properties. In addition, the SNpr contains smaller populations of other neurons, including glutamatergic neurons. Here, we discuss regionalization of the SNpr, in particular the division of the SNpr neurons to anterior (aSNpr) and posterior (pSNpr) subtypes, which display differences in many of their features. We hypothesize that unique developmental and molecular characteristics of the SNpr neuron subtypes correlate with both region-specific connections and notable functional specializations of the SNpr. Variation in both the genetic control of the SNpr neuron development as well as signals regulating cell migration and axon guidance may contribute to the functional diversity of the SNpr neurons. Therefore, insights into the various aspects of differentiation of the SNpr neurons can increase our understanding of fundamental brain functions and their defects in neurological and psychiatric disorders, including movement and mood disorders, as well as epilepsy.

## Introduction

Basal ganglia, a network of brain structures and nuclei in the forebrain and anterior brainstem, evaluate external and internal signals and use this information to select, activate and invigorate appropriate behavior, often including voluntary movement (Dudman and Krakauer, 2016; Klaus et al., 2019). These brain functions are of paramount importance for the fitness of an organism and the various underlying cell types and circuits have aggregated to form the basal ganglia system early in the evolution (Grillner and Robertson, 2016).

**FIGURE 1:**
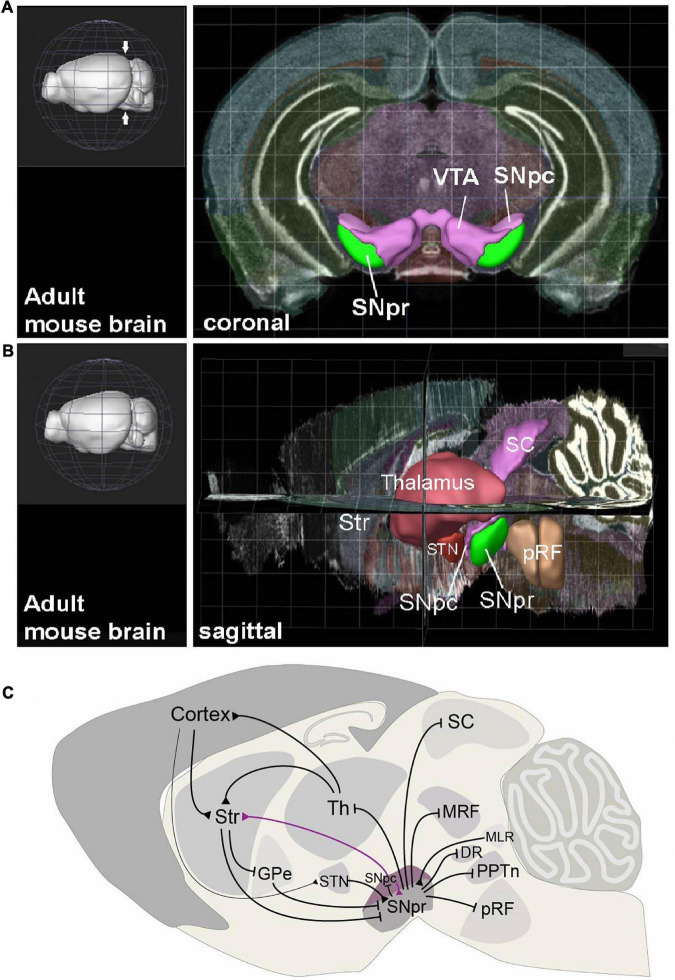
The anatomical context and connectivity of Substantia Nigra. **(A)** Coronal view to the adult mouse brain at the level on midbrain (level indicated with arrows on the 3D image in left). The Substantia Nigra pars reticulata (SNpr), pars compacta (SNpc) and Ventral Tegmental Area (VTA) are highlighted. **(B)** Sagittal view to adult mouse brain, showing the positions of SNpr and its interconnected structures. Str, striatum; STN, Subthalamic nucleus; SC, Superior colliculus; pRF, pontine reticular formation. **(C)** Input and output structures of the SNpr and their interconnections in mature mouse brain. Black arrows represent inhibitory GABAergic (blunt arrowhead) and excitatory glutamatergic (triangular arrowhead) projections. The dopaminergic neuron projection from SNpc to striatum (Str) is shown in violet. Th, Thalamus; GPe, Globus Pallidus, external segment; MRF, midbrain reticular formation; MLR, midbrain locomotor region; DR, Dorsal Raphe nucleus; PPTn, pedunculopontine tegmental nucleus. The views in **(A,B)** are created using the Brain Explorer 2 app (Allen Institute for Brain Science, https://mouse.brain-map.org/static/brainexplorer).

The basal ganglia include separate structures for information input, modulation and output. The main input areas sending afferent projections to the basal ganglia are the striatum and the subthalamic nucleus (STN), which in turn receive projections from the cortex and thalamic nuclei (Figure 1C). The modulatory components include the dopaminergic nuclei in the ventral midbrain. These structures and their incorporation to the basal ganglia network have been extensively reviewed earlier (Deniau et al., 2007; Takakusaki, 2008; Grillner and Robertson, 2016; Hikosaka et al., 2019; Klaus et al., 2019; Arber and Costa, 2022). The output of the basal ganglia is funneled through two brain centers: the internal segment of Globus Pallidus (GPi) and Substantia Nigra pars reticulata (SNpr). These nuclei contain GABAergic projection neurons that tonically inhibit their targets in the dorsal midbrain, anterior brainstem and thalamus, to control activation of movement and other aspects of behavior. Thus, the basal ganglia participate in the control of behavior by disinhibition: upstream components of the basal ganglia network suppress the GPi and SNpr, resulting in activation of the GPi and SNpr targets under excitatory cortical stimulation, leading to the effective behaviors (Hikosaka, 2007). This model has extensive experimental support, but the SNpr neuron function also appears more complex. While the activity of some SNpr neurons decreases during the activation of behavior, many others increase their firing, suggesting that in addition to allowing the desired behavior, the SNpr also suppresses competing actions (Mink, 1996; Gulley et al., 2002; Meyer-Luehmann et al., 2002). Furthermore, recent studies suggest that disinhibition may not be the only operational mechanism of the SNpr, and that the SNpr neurons can also elicit activation of their target neurons by rebound depolarization (Villalobos and Basso, 2022). In addition, there also are some excitatory glutamatergic neurons in the SNpr (Antal et al., 2014; Morales and Root, 2014). Importantly, both earlier and recent studies promote a view that to regulate various types of behaviors, the SNpr is divided into discrete modules, differing in their connectivity and function (Deniau et al., 2007; McElvain et al., 2021).

How the basal ganglia output is controlled by specialized SNpr neurons, responsible for distinct behavioral outcomes, remains incompletely understood. A prerequisite for understanding the cell type-specific output is to have information on the properties and diversity of the output neurons at multiple levels (Arber and Costa, 2022). In this review, we focus on the cellular composition of the SNpr. We discuss the embryonic development of the molecularly distinct subtypes of SNpr neurons, and give an overview of information available about their connectivity patterns as well as region-specific functions. The focus of this review is on the possible correlations between the neuronal subtype-specific developmental, neuroanatomical and functional features, which have remained largely overlooked to date. Insights to the SNpr composition and function are central for understanding fundamental aspects of behavioral regulation and have implications for understanding several brain diseases such as movement disorders, attention deficit and hyperactivity, as well as epilepsy.

## The Substantia Nigra pars reticulata neurons and their development

The SNpr is an anatomically easily recognizable nucleus in the most ventral brainstem, next to the dopaminergic neurons in the Substantia Nigra pars compacta (SNpc) and Ventral Tegmental Area (VTA) (Figure 1A). In the mouse, the SNpr extends from the posterior diencephalon to the midbrain, with its posterior limit at the midbrain-hindbrain border (Figure 1B). The majority of the SNpr neurons are inhibitory GABAergic neurons, but the SNpr also contains glutamatergic and dopaminergic neurons, as well as neurons using both glutamate and dopamine as neurotransmitters (Nair-Roberts et al., 2008; Yamaguchi et al., 2013; Antal et al., 2014). In addition, few cholinergic neurons are also located in the posterior SNpr (Gould and Butcher, 1986). The SNpr neurons are mostly projection neurons and local interneurons appear very few in number (Deniau et al., 2007). Most of the research discussed here has focused on the GABAergic component of the SNpr, although some of the SNpr glutamatergic neurons may be both developmentally and functionally related to these neurons.

### Developmental origins of the Substantia Nigra pars reticulata GABAergic neurons

**FIGURE 2:**
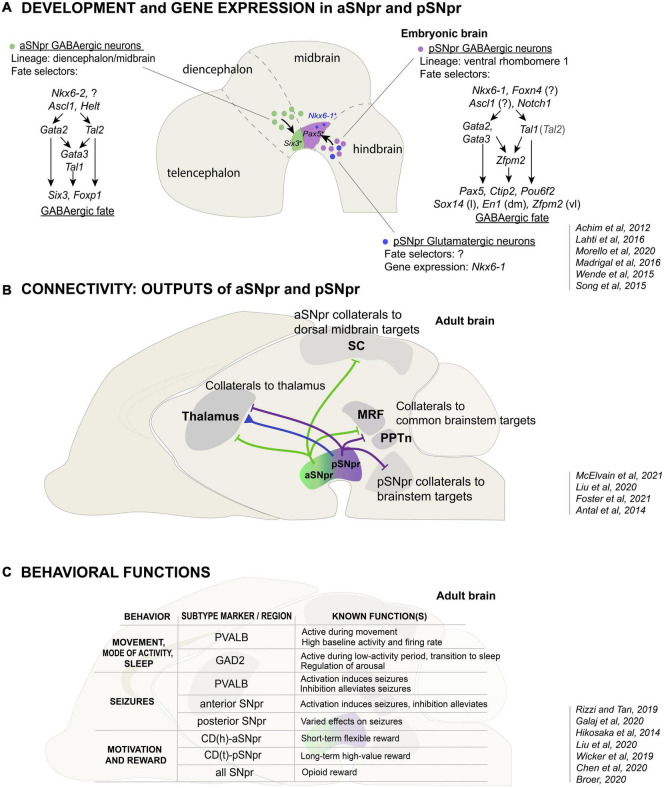
The development, connectivity and function of the anterior and posterior SNpr neurons. **(A)** Developmental origin and differential gene expression in the aSNpr GABAergic neurons and the pSNpr GABAergic and glutamatergic neurons. The aSNpr GABAergic neurons originate in the *Nkx6-2* expressing neuronal progenitors in the ventrolateral midbrain or posterior diencephalon (green), and are characterized by the expression of *Six3* and *Foxp1*. aSNpr neurons require the function of the proneural genes *Ascl1* and *Helt* and the selector genes *Tal1* and *Gata2* in order to acquire GABAergic neuron phenotype. In early precursors, *Gata2* and *Tal2* are activated independently of each other and can have both common and separate target genes. The pSNpr GABAergic neurons (violet) and glutamatergic neurons (blue) originate in the *Nkx6-1* expressing neuronal progenitors in the anterior ventrolateral hindbrain. pSNpr progenitors express *Foxn4*, *Ascl1* and *Notch1*. pSNpr GABAergic neurons are characterized by the expression of *Pax5, Ctip2* and *Pou6f2*, and require the function of *Tal1* and *Gata2/Gata3* to acquire GABAergic neuron phenotype. *Sox14*, *En1* and *Zfpm1* expression marks further subtypes of pSNpr neurons, which are located in distinct spatial positions: lateral (l), dorsomedial (dm) or ventrolateral (vl) pSNpr. The transcription factors involved in the fate specification of pSNpr glutamatergic neurons are not known. The relatively minor populations of dopaminergic and cholinergic SNpr neurons are not shown. **(B)** Differences in the output connectivity of aSNpr and pSNpr neurons. One of the hallmarks of SNpr neurons are the axon-collaterals. The common targets of the aSNpr and pSNpr are thalamus, MRF (midbrain and pontine reticular formation) and PPTn (pedunculopontine nucleus. In addition to projections to the common targets, the aSNpr neuron axon-collaterals (green) project to superior colliculus (SC), while the pSNpr axon-collaterals (violet) more often project to brainstem. Thalamus is one of the major targets of the SNpr glutamatergic neurons (blue). SNpr glutamatergic neurons specifically project to the nucleus reticularis of thalamus (nRT, see text). **(C)** Variety of functions of the SNpr neuron subtypes. The table summarizes the known functions of the *Pvalb*- or *Gad2*-expressing SNpr GABAergic neurons in the control of motor activity and behavioral state, and the functions of the anterior and posterior parts of the SNpr in the seizure activity, motivation, and reward. How these functions are related to specific molecularly defined SNpr neuron subtypes remains to be shown.

Although located primarily in the ventral midbrain in the mature brain, the SNpr GABAergic neurons are largely derived outside the embryonic midbrain neuroepithelium. The first indication of this was the observation that abundant SNpr GABAergic neurons were still present in mouse mutants where the midbrain GABAergic neurogenesis completely failed (Kala et al., 2009). Cre-recombinase driven fate-mapping in the mouse subsequently indicated an origin for some SNpr GABAergic neurons in the hindbrain (Achim et al., 2012). Importantly, a hindbrain-specific Cre-driver (*Gbx2*^*CreERT*2^) labeled the posterior part of the SNpr (pSNpr), but not its anterior part (aSNpr). Studies of the development of GABAergic neuron subtypes in the rhombomere 1 (r1) of the anterior hindbrain have further refined the origin of the pSNpr neurons in the ventrolateral r1 neuroepithelium expressing the homeodomain transcription factor (TF) NKX6-1, and in particular the anterior part of this neuroepithelial region close to the midbrain-hindbrain border (Morello et al., 2020a; Figure 2A). On the other hand, fate mapping with the *Nkx6-2*^*CreERT*2^ –driver suggested that the aSNpr neurons are derived from the *Nkx6-2* expressing neuronal progenitors or precursors found in the ventrolateral midbrain-diencephalon region (Qiu et al., 1998; Moreno-Bravo et al., 2010; Madrigal et al., 2016). The exact origin of aSNpr neurons remains to be established with regionally more restricted progenitor cell labeling (Qiu et al., 1998; Moreno-Bravo et al., 2010; Madrigal et al., 2016).

In summary, the SNpr is divided into two developmentally independent GABAergic components, termed aSNpr and pSNpr in this review, each of which originates in distinct antero-posterior brain compartments (Figure 2A). The ventrolateral neuroepithelial domains giving rise to the aSNpr and pSNpr neurons are marked by the expression of specific homeodomain TFs. Interestingly, in addition to the GABAergic neurons of the pSNpr, the precursors in the ventrolateral r1 also produce developmentally related glutamatergic neuron types, some of which may also contribute to the SNpr (Figure 2A, blue).

### Regulation of the differentiation of the Substantia Nigra pars reticulata GABAergic neurons

GABAergic neurogenesis, production of post-mitotic GABAergic neuron precursors from proliferative progenitor cells, is regulated by different proneural TFs in the diencephalon/midbrain and the hindbrain, giving rise to aSNpr and pSNpr, respectively. In the diencephalon/midbrain *Ascl1* and *Helt* are required for GABAergic precursor production (Song et al., 2015; Wende et al., 2015). In turn, similar to spinal cord, GABAergic neurogenesis in the ventrolateral hindbrain may be controlled by *Foxn4* and *Ascl1* (Li et al., 2005; Misra et al., 2014; Figure 2A).

The differentiation of postmitotic pSNpr and aSNpr neurons is guided by related, but somewhat different sets of TFs. Members of the GATA zinc finger TF family, GATA2 and GATA3, as well as members of the Tal/Scl bHLH TF family, TAL1 and TAL2, interact in a heteromeric TF complex and are important for the acquisition of GABAergic neuron identity in the spinal cord, r1 and midbrain (Zhou et al., 2000; Joshi et al., 2009; Kala et al., 2009; Achim et al., 2012, 2013; Porcher et al., 2017). These TF genes act as developmental selector genes (Hobert and Kratsios, 2019). The differentiation of the pSNpr neurons is regulated by *Tal1*, *Gata2*, and *Gata3*, the latter two functioning redundantly (Achim et al., 2012; Lahti et al., 2016; Figure 2A). During the embryonic development, most of the pSNpr GABAergic neuron precursors exit the cell cycle between E11.5-E12.5, slightly after the GABAergic neurons of the midbrain reticular formation, but before the GABAergic neurons of the dorsal midbrain (superior colliculi) (Achim et al., 2012). The expression of *Tal1*, *Gata2* and *Gata3* is robustly activated at this stage in the neuronal precursors of the ventrolateral r1 as these cells exit the cell cycle and migrate out of the neuroepithelium. At later stages, *Gata2* expression decreases while *Gata3* expression is maintained in the GABAergic neurons. Importantly, the ventrolateral r1 comprises *Nkx6-1* expressing progenitors that can give rise to post-mitotic precursors differentiating into either GABAergic or glutamatergic neurons. TAL1, GATA2, and GATA3 act as selectors of the GABAergic identity, preventing the alternative glutamatergic differentiation (Lahti et al., 2016). It is also known that in the pSNpr precursors, *Gata2* and *Gata3* genes are activated independent of each other and either one of them is required for GABAergic differentiation. In turn, the expression of *Nkx6-1* is maintained only in the differentiating glutamatergic precursors. Asymmetric Notch signaling, probably mediated by NOTCH1 receptor and its ligands DLL3 and DLL4, is associated with the activation of selector gene expression and GABAergic fate acquisition in the post-mitotic precursors in the ventrolateral r1 (Morello et al., 2020a). Similar role for NOTCH1-DLL4 signaling has been demonstrated in the ventrolateral V2 region of the spinal cord (Del Barrio et al., 2007; Peng et al., 2007).

The differentiation of the aSNpr GABAergic neurons is also initiated by *Gata2* function, *Gata2* being the main selector gene for GABAergic identity in early postmitotic precursors in the midbrain and posterior diencephalon (P1–P2) (Kala et al., 2009; Achim et al., 2012; Virolainen et al., 2012). In contrast to the pSNpr precursors where *Gata2* and *Gata3* operate redundantly, *Gata2* function is essential for *Gata3* expression in the midbrain and diencephalon, and inactivation of *Gata2* alone is sufficient to prevent GABAergic differentiation of the aSNpr precursors (Achim et al., 2012; Lahti et al., 2016). Furthermore, unlike in the pSNpr precursors, *Tal1* is not required for the development of aSNpr precursors, where the requirement of a bHLH selector function may be compensated by a related TF TAL2 (Achim et al., 2013).

Thus, the early precursors of the pSNpr and aSNpr neurons are dependent on different combinations of GATA and TAL TFs that function as GABAergic fate selectors.

### Molecular subtypes of the Substantia Nigra pars reticulata GABAergic neurons

The SNpr GABAergic neurons are heterogeneous in their cytoarchitecture and expression of neurochemical markers, including Parvalbumin (PVALB), Calretinin, and Nitric oxide synthase, with the subtypes differing in their spatial distribution along the medio-lateral and antero-posterior axes of the SNpr (Gonzalez-Hernandez and Rodriguez, 2000; Lee and Tepper, 2007).

The developmental regulation leads to a unique molecular profile in a cell or cell type, defining its functional properties. Both pSNpr and aSNpr neurons robustly express genes required for the GABAergic neurotransmission, such as Glutamic acid decarboxylase 1 (*Gad1*), and markers of the GABAergic neurons in the posterior diencephalon-midbrain-ventral hindbrain region, such as *Gata3* (Lahti et al., 2016). However, the developmentally distinct SNpr neuron populations also differ in other TF gene expression. For example, the pSNpr neurons express TF genes *Pax5*, *Ctip2*, and *Pou6f2*, whereas the aSNpr neurons express *Six3* and *Foxp1* (Achim et al., 2013; Lahti et al., 2016; Madrigal et al., 2016; Saunders et al., 2018; Morello et al., 2020a). Developmentally, the pSNpr neurons are likely derived from the *Pax5* expressing precursors located close to the midbrain-hindbrain border in the ventrolateral r1. The early regulation of differentiation and *Pax5* expression in the r1 precursors is probably subject to the antero-posterior patterning and other signals from the midbrain-hindbrain/isthmic organizer (Wurst and Bally-Cuif, 2001).

Besides the two main SNpr neuron subtypes, aSNpr and pSNpr, there is further molecular heterogeneity in the SNpr, especially among the pSNpr neurons. For example, the most lateral pSNpr is distinguished by *Sox14* expression, and *En1* and *Zfpm2* expression marks additional dorsomedial and ventrolateral pSNpr neuron subtypes (Lahti et al., 2016; Saunders et al., 2018; Zeisel et al., 2018). Anatomically, the molecularly distinct aSNpr and pSNpr neurons are clearly segregated in the embryonic brain (Lahti et al., 2016; Morello et al., 2020a) and despite partial intermingling during later development, the aSNpr and pSNpr neurons retain their regional bias and can be identified based on subtype-specific gene expression in the adult brain.

The distinct TF expression profiles described above likely manifest in the expression of subtype-specific gene products that determine neuronal function, but to date, this remains incompletely understood. Interestingly, the calcium-binding protein PVALB, used for mapping the SNpr connections and functions (see below), is preferentially, although not exclusively, expressed in the *Foxp1* positive putative aSNpr neurons and the lateral SNpr (Saunders et al., 2018; Liu et al., 2020). Also GAD2, an enzyme involved in GABA biosynthesis, is expressed at different levels in several SNpr GABAergic neuron subpopulations and, in contrast to PVALB, its expression is enriched in the medial part of the SNpr (Liu et al., 2020).

In summary, the SNpr GABAergic neurons are molecularly heterogeneous. The subtypes of mature SNpr neurons that differ in their developmental origins and regulation can also be distinguished by differential gene expression (Figure 2A). Recent scRNAseq studies (La Manno et al., 2016; Lutas et al., 2016; Saunders et al., 2018; Agarwal et al., 2020; Morello et al., 2020a) have contributed to the understanding of the molecular features and subtypes among the SNpr neurons, but the full resolution seems to not be achieved yet.

### Migration of the Substantia Nigra pars reticulata GABAergic neuron precursors

The later developmental mechanisms, including the molecular cues guiding the migration of the SNpr GABAergic precursors to their correct location in the ventral midbrain are beginning to be elucidated. The SNpr GABAergic neurons move to the ventral midbrain mostly between E14.5-E16.5 (Achim et al., 2012; Vasudevan et al., 2012; Brignani et al., 2020). In the ventral midbrain the GABAergic neurons are preceded by the SNpc and VTA dopaminergic neurons, but different conclusions have been reached considering the role of dopaminergic neurons in the guidance of GABAergic precursor migration (Vasudevan et al., 2012; Brignani et al., 2020). Several cell adhesion and migration associated proteins, such as DCC, Netrin-1 (NTN1), Ephrins and Plexins are expressed across the SNpr area. Furthermore, NTN1, released by striatal axons in the cerebral peduncle crossing the ventral midbrain, is required for the migration of SIX3^+^ aSNpr neurons to their location ventral to SNpc dopaminergic neurons (Brignani et al., 2020). The aSNpr neurons express *Dcc*, *Neogenin* and *Dscam*, of which DSCAM is the main functional NTN1 receptor in migrating aSNpr precursors (Brignani et al., 2020). Importantly, in contrast to the aSNpr precursors, migration of the GABAergic precursors of the posterior SNpr is not affected by depletion of the NTN1 expression in the striatal axons. The specific mechanisms guiding the pSNpr neuron migration remain less understood. A guidance molecule EPHB1 is expressed in the SNpr GABAergic neurons during development, and *Ephb1* mutant mice show reduced numbers of SNpr cells at both anterior and posterior levels, and display locomotor hyperactivity (Richards et al., 2007). How the dopaminergic and GABAergic neurons interact in the ventral midbrain to form the SNpc and SNpr components of the Substantia Nigra still remains an open question.

### Development and molecular subtypes of the other Substantia Nigra pars reticulata neurons

Compared to the GABAergic neurons, less is known about the other neuron types in SNpr, including glutamatergic neurons, and the neurons co-releasing glutamate and dopamine. Many SNpr glutamatergic neurons express the TF gene *Nkx6-1* (Lahti et al., 2016; Saunders et al., 2018) and are located in the dorsomedial and posterior regions of the SNpr (Antal et al., 2014; Morales and Root, 2014; Figures 2A,B). Similar to the pSNpr GABAergic neurons, these neurons likely have their embryonic origin in the ventrolateral r1, as their numbers are markedly increased by inactivation of *Tal1* or *Gata2/3* and the resulting GABAergic-to-glutamatergic fate transformation of the r1 precursors (Lahti et al., 2016). These NKX6-1 positive neurons may represent the SNpr glutamatergic neurons that do not co-express dopaminergic neuron characteristics (Antal et al., 2014) (see below). The SNpr neurons co-releasing dopamine and glutamate seem to be molecularly and developmentally related to the SNpc and VTA dopamine neuron subgroups (Poulin et al., 2020).

## Connections of the Substantia Nigra pars reticulata neurons and their regional specification

### Input to the Substantia Nigra pars reticulata GABAergic neurons

The SNpr receives GABAergic input from the striatum and the external segment of Globus Pallidus (GPe), and glutamatergic input from the STN and a subset of neurons in the midbrain locomotor region (MLR) (the RBP4 expressing glutamatergic neurons in the midbrain reticular formation) (Grillner and Robertson, 2016; Ferreira-Pinto et al., 2021; Arber and Costa, 2022). The striatal input to the SNpr is organized into the direct inhibitory pathway, and the indirect excitatory pathway through the STN (Kravitz et al., 2010). The direct striatal and indirect striatopallidal inputs converge in SNpr, maintaining the features of the striatal topographical organization as demonstrated by systematic tracing studies in rodents (Deniau et al., 1996, 2007; Lee et al., 2020; Foster et al., 2021). In the SNpr, the striatal input is directed to at least six identifiable longitudinal columns spanning the entire antero-posterior extent of the SNpr. Thus, the columns receiving information from the striatum do not strictly correlate with the developmental aSNpr/pSNpr division. Instead, distinct striatal areas preferentially connect to different medio-lateral regions in the SNpr: the dorsolateral striatum connecting to more lateral SNpr and ventromedial striatum to more medial SNpr, to regulate various aspects of movement (Deniau et al., 2007; Lee et al., 2020). Basal ganglia nuclei, including the striatum, GPe and STN, were found to be the main source of input to both *Pvalb*- and *Gad2*-expressing SNpr neurons. Compared to the *Pvalb*-expressing neurons, the *Gad2*-expressing neurons receive more variable inputs, including connections from the anterior brainstem regions implicated in the regulation of both movement and brain state, such as SC, MLR, dorsal raphe (DR), and periaqueductal gray (PAG) (Liu et al., 2020).

The striatal input to the SNpr is also topographically organized in primates. The dorsal striatum in primates is composed of two nuclei, the Caudate and the Putamen. Of these, the Putamen projects primarily to the GPi, whereas the main target of the Caudate nucleus is the SNpr (Parent et al., 1984; Smith and Parent, 1986). The Caudate nucleus is further divided into sub-regions that project to non-overlapping targets in the SNpr and have been shown to be preferentially activated during distinct assays of object recognition in primates. The projections from the head of the Caudate nucleus CD(h) are directed toward the anterior (rostro-ventral-medial) region of the SNpr, whereas the tail of the Caudate nucleus CD(t) projects to the posterior (caudal-dorsal-lateral) SNpr (Hikosaka et al., 2014; Yasuda and Hikosaka, 2015).

In addition to these inhibitory and excitatory projections, the SNpr receives dopamine released by the dendrites of adjacent SNpc dopaminergic neurons (Cheramy et al., 1981; Levey et al., 1993). SNpr GABAergic neurons express dopamine receptors D1R and D5R, and the direct dopamine signaling might contribute to tonic firing of SNpr neurons (Zhou et al., 2009). In addition to the SNpr neurons, dopamine may affect local astrocytes, which strongly express D1R in the SNpr (Nagatomo et al., 2017). As discussed above, the SNpr neurons receive extensive indirect dopamine regulation *via* striatonigral GABAergic projection neurons, which also express D1R.

### Output from the Substantia Nigra pars reticulata GABAergic neurons

The SNpr sends GABAergic output to the dorsal midbrain (the superior colliculi, SC, and the inferior colliculi, IC), brainstem nuclei and the thalamus (Deniau and Chevalier, 1992). This output affects downstream targets regulating behavior and may provide feedback and efference copy information to the upstream basal ganglia structures, the cortex and to the SNpr itself. Systematic anterograde mapping of the SNpr projections revealed 42 different output sites of the SNpr, including several targets in the brainstem region (McElvain et al., 2021). Among these targets, the relative SNpr output varies greatly, two-thirds of it being directed to the brainstem reticular formation and the colliculi. One prominent feature of the SNpr output are the axon collaterals. Using the axon-collaterals, the SNpr neurons can have both specific targets, for example in the dorsal midbrain and the brainstem, as well as shared targets in thalamus, pedunculopontine nucleus (PPTn) and midbrain reticular formation (Liu et al., 2020; McElvain et al., 2021). There are also non-collateralising SNpr neuron populations, such as the projections to pontine and midbrain reticular formation.

The SNpr projections to the dorsal midbrain and the brainstem send information out of the basal ganglia loop circuits toward action initiation. Interestingly, SNpr neurons projecting to specific targets in the dorsal midbrain and the brainstem are spatially clustered, showing enrichment in different areas of SNpr. The neurons sending projections to the superior and inferior colliculi (SC and IC) in the dorsal midbrain are located in the anterolateral SNpr, whereas the neurons sending projections to the brainstem nuclei in the hindbrain, including the dorsal raphe and pontomedullary reticular formation, are located in the posteromedial SNpr (Deniau and Chevalier, 1992; McElvain et al., 2021; Figure 2B). This is further supported by distinct projection patterns: lateral *Pvalb*-expressing neurons are connected to the SC and MLR, brain regions related to locomotion, while medial *Gad2*-expressing SNpr neurons project more widely to brainstem regions involved in brain-state regulation (Saunders et al., 2018; Liu et al., 2020). It is possible that these differences in the regional projection patterns are corollary to the distinct molecular properties and developmental histories of the inherent neuron subtypes. Interestingly, the origin of the pSNpr neurons in the embryonic r1 parallels the projection of the posterior and medial SNpr neurons to the brainstem nuclei located in the hindbrain. It is thus possible that the translocation of the cell bodies of the pSNpr neurons is associated with establishment of their connectivity, as seen in some other neuronal subtypes in the developing brain (Lambert de Rouvroit and Goffinet, 2001).

In addition to SNpr neurons having region-specific output targets, there is regional specification also within the shared targets. In particular, most SNpr neurons are characterized by a projection to the thalamus. The target areas in the thalamus, however, differ between the SNpr neurons connected to the dorsal midbrain and the brainstem: the SNpr neurons projecting to the dorsal midbrain send collaterals to the more lateral thalamus, whereas the SNpr neurons projecting to brainstem send collaterals to the more medial regions of the motor and intralaminar thalamic nuclei (McElvain et al., 2021). These connections appear to be involved in parallel closed-loop circuits between the cortex, striatum, SNpr and thalamus (Foster et al., 2021). Thus, the SNpr contains parallel modules that can affect both ascending and descending targets regulating behavior.

As mentioned above, the SNpr is thought to contain only sparse local interneurons. Rather than local interneurons, the axon-collaterals of the SNpr projection neurons provide feedback and mutual inhibitory interactions within the SNpr, as well as project to the adjacent dopaminergic SNpc (Tepper et al., 1995; Mailly et al., 2003; Lee and Tepper, 2007; Brown et al., 2014; Galaj et al., 2020). The SNpr neurons projecting to the SNpc are characterized with a relatively high level of PVALB expression (Rizzi and Tan, 2019).

In summary, the SNpr receives region-specific input and is a source of region-specific output. The regional organization of the input and output show similarities, suggesting separate information channels (Deniau et al., 2007), but the regional maps may not fully overlap, providing some input integration capacity. Although there are clear differences in the connectivity along the antero-posterior, dorso-ventral and medio-lateral axes of the SNpr, the extent of correlation between the distinct projection patterns and the molecularly defined SNpr neuron subtypes is not yet shown.

### Connections of the Substantia Nigra pars reticulata glutamatergic neurons

Similar to the SNpr GABAergic neurons, the SNpr glutamatergic neurons also receive inhibitory input from the striatum and project to the thalamus (Antal et al., 2014; Figure 2B). However, the thalamic targets of the GABAergic and glutamatergic neurons differ. Whereas the SNpr GABAergic neurons provide inhibitory input to the ventral thalamic area (the ventroposterolateral nucleus), the glutamatergic projection provides excitatory input to the nucleus reticularis of thalamus (nRT), and both GABAergic and glutamatergic neurons project to the posterior nucleus group (Antal et al., 2014). The specific function of the SNpr glutamatergic neurons is unknown.

## Signaling properties of the Substantia Nigra pars reticulata neurons

The SNpr GABAergic neurons are spontaneously active suppressing their targets. They display a broad range of activity characteristics. Importantly, the topographically organized SNpr neuron subtypes, differing in their projections to the collicular and brainstem targets, are also specialized in their intrinsic electrophysiological properties (McElvain et al., 2021). The neurons projecting to the lateral and central SC, involved in sensorimotor function, show higher level of activity and narrow action potentials, whereas the projection neurons to the neuromodulatory dorsal raphe are the slowest population. These electrophysiological specializations further support the existence of molecularly and functionally segregated SNpr neuron subtypes. Consistent with this, the *Pvalb*-expressing SNpr neurons, were also shown to fire at higher frequencies and have higher baseline activity than the *Gad2*- and *Vgat* (*Slc32a1*) expressing SNpr neurons (Rizzi and Tan, 2019; Liu et al., 2020; Figure 2C). Recent studies have addressed fine aspects of the ion channel expression and function in the SNpr GABAergic neurons (Zhou et al., 2009; Zhou and Lee, 2011; Lutas et al., 2016), demonstrating notable diversity in ion channel gene expression, but this information has not been linked to spatial positions. Those findings could, in future, be carefully compared with the recent single-cell sequencing data. One especially promising approach here would be to use spatial transcriptomics studies as reference.

## Behavioral functions of the Substantia Nigra pars reticulata neurons

### Movement, action selection and decision making

The hallmark feature of the basal ganglia output is the inhibition of movement, combined with selective activation of a movement by disinhibition when a tonic inhibitory output from the SNpr is paused (Chevalier and Deniau, 1990). This principle may be extended to selection of series of movements, behaviors and behavioral patterns. Different regions of the SNpr appear to regulate different types of movement, such as orofacial movement (licking) or body movements (turning), as suggested by the topographically organized projections from the striatum to the SNpr, and the behavioral consequences of stimulation of distinct striatal regions (Lee et al., 2020). The SNpr GABAergic neurons are thought to integrate “Go” signals (inhibitory direct input from the striatum) and “Stop” signals (excitatory input from the STN, a target of the striatal indirect pathway, and MLR) (Ferreira-Pinto et al., 2021). Activation of both the *Pvalb*-expressing and the *Gad2*-expressing neurons suppresses movement (Liu et al., 2020). However, during normal behavior, the *Pvalb*-expressing neurons have been shown to be more active during high motor activity, which is thought to reflect their function in suppression of unwanted movements (Liu et al., 2020). The pathways involving medial or lateral SNpr may affect movement differently, and earlier studies have indeed suggested that the lateral and medial regions of the SNpr differentially control locomotion and postural muscle tone (Takakusaki et al., 2003). Interestingly, the SNpr contains a specific region enriched in neurons that are associated with correct “Stop” signaling (Schmidt et al., 2013). This “hotspot” of “Stop” signaling is located in the dorsolateral SNpr and may comprise specific aSNpr/pSNpr neurons subgroups, such as the *Sox14* positive lateral pSNpr neurons (Lahti et al., 2016).

### Mode of activity and sleep

The SNpr also controls transitions between behavioral states, such as different levels of motor activity and sleep (Figure 2C). In particular, the *Gad2*-expressing neurons in the medial SNpr have been shown to fire more during behavioral states of low motor activity, immobility, and sleep. Furthermore, activation of the *Gad2*-expressing cells, in addition to suppression of movement, also induced behavioral transition to sleep. This is in contrast to the *Pvalb*-expressing cells, whose activation suppressed movement but had no direct effect on sleep. Consistently, inactivation of the *Gad2*-expressing neurons more strongly inhibited sleep. These findings correlate with the projection of the *Gad2*-expressing cells to the brainstem neuromodulatory centers, including dorsal raphe and locus coeruleus, which regulate the behavioral state (Liu et al., 2020) (see above).

### Seizures

Perhaps related to its role in action selection, the SNpr function has been associated with the control of seizures. Already 40 years ago, it was found that the injection of GABA agonist muscimol into the SNpr has anticonvulsant effects (Iadarola and Gale, 1982). Interestingly, multiple studies have reported anterior-posterior differences in how SNpr is involved in seizure control. The anterior and posterior SNpr are differentially active during seizures (Gernert et al., 2004; Veliskova et al., 2005). Moreover, modulation of neuronal activity in distinct regions of the SNpr have different effects on seizure predisposition. Several studies have found pharmacological suppression of the anterior SNpr to be anticonvulsive, whereas suppression of the posterior SNpr has been reported to have both anticonvulsive and proconvulsive effects (Broer, 2020; Figure 2C). Consistent with this, opto- or chemogenetic activation of the *Pvalb*-expressing SNpr neurons was found to amplify seizures, whereas their inhibition alleviated seizure activity (Chen et al., 2020). Optogenetic inhibition of the GABAergic neurons in the anterior SNpr neurons was also found to suppress different kinds of seizures in rats (Wicker et al., 2019). Furthermore, suppression of the SNpr projections to the SC recapitulated these effects. In contrast, suppression of the SNpr projection to the PPTn, a target of the posterior SNpr, had more varied effects and even amplified some of the seizure types (Wicker et al., 2019). Thus, the anterior and posterior regions of the SNpr, and their region-specific output targets, have distinct roles in the control of seizures.

### Reward

In addition to the GABAergic and dopaminergic neurons in the VTA that have been extensively studied in the context of reward, the SNpr GABAergic neurons have also been shown to mediate avoidance and reward function, and are especially important for opioid action (Rizzi and Tan, 2019; Galaj et al., 2020). Compared to the VTA, the SNpr GABAergic neurons more abundantly express μ-opioid receptors and their optogenetic activation more potently inhibits opioid reward. These neurons may be related to the *Pvalb*-expressing SNpr GABAergic neurons that project to the adjacent SNpc and regulate both movement and reward (Rizzi and Tan, 2019).

The function of basal ganglia and SNpr in value-based behavior and decision making has been elucidated by the studies in primates. Interestingly, different roles have been assigned to the anterior and posterior parts of the SNpr that receive differential innervation from the CD(h) and CD(t) (see above). In primates, the activity of the CD(t)-pSNpr circuit was specifically observed during the saccade toward previously learned high-value objects, while the activity of the CD(h)-aSNpr circuit was associated with the selection of objects with short-term, changing value (Hikosaka et al., 2014; Yasuda and Hikosaka, 2015; Figure 2C).

## Conclusion and relevance for brain disease

A number of recent studies have addressed the region-specific functions of the SNpr neurons in the context of selecting appropriate behavior, initiation of specific movements and the regulation of behavioral states. The regional differences and specific functions are likely to match the molecularly distinct cell types, as demonstrated by specific manipulation of the activity of the *Pvalb*- and *Gad2*-expressing SNpr neurons (Figure 2C). In addition to acute optogenetic manipulation, behavioral studies of animal models with defects in differentiation of specific SNpr neuron subtypes would be important for understanding behavioral disorders, which often have a developmental basis. For example, mice carrying a mutation in the *Tal1* gene lack pSNpr neurons and show ADHD-like behaviors (Morello et al., 2020b). However, these mice also have defects in other brainstem nuclei, underlining the importance of finding strategies for more specific manipulation of the SNpr cell types.

SNpr has been shown to gate different types of behaviors, and studies reviewed here have addressed its broad (for example controlling the state of behavioral activity) as well as more focused (for example activating a specific movement) functions. The functional repertoire of SNpr appears to be based on distinct SNpr neuron subtypes and their cell-type specific connectivity that form parallel, segregated pathways regulating specific behaviors (Lee et al., 2020). We have discussed here how the SNpr neurons differ by their development, gene expression, connectivity and functions. We propose existence of two main categories of the SNpr GABAergic neurons, the anterolateral aSNpr and posteromedial pSNpr neurons, the features of which reflect their distinct developmental histories in the embryonic brain and suggest that the functional differences of these main SNpr neuron subtypes are intrinsically defined already in the early precursors. It appears that this division represents just the first level of SNpr GABAergic neuron heterogeneity and that both aSNpr and pSNpr contain diverse subcategories, in particular along their medio-lateral axis. This heterogeneity can arise from intrinsic differences in the early precursors, or later extrinsic influences from the neuronal environment and circuitry. Although both aspects likely contribute to the diversity, the fact that molecularly distinct pSNpr neuron subtypes are found in the embryonic brain suggests that at least to some extent their differences are determined early during precursor differentiation. It should be noted that anatomical correlates, although suggestive, are only first approximations, and future studies using cell-type-specific labeling should directly test how the regional differences in the various properties of the SNpr neurons are related to each other. Better understanding of the SNpr neurons also requires more focused and deeper gene expression profiling. This can be expected to reveal SNpr neuron subtypes and their specific markers that would be useful tools for mapping SNpr neuron input and output connections, patterns of activity, and physiological functions in the regulation of behavior. Thus, developmental and genetic studies are expected to guide deeper analyses of the SNpr neurons by viral tracing, electrophysiological techniques, and optogenetic methods.

As a center for behavioral gating, understanding the SNpr has broad implications for brain disease (Figure 2C). A defect in the control of the activity state is a fundamental characteristic of the sleep disorders and attention deficit hyperactivity disorders. Epilepsy reflects a failure to control the overall brain activity pattern and to carry out normal action selection process resulting in seizures (Salpekar, 2018; Broer, 2020). Movement disorders involve more focused defects in the activation of motor activity, and GABAergic basal ganglia output is thought to be overactive in Parkinson’s disease (Wichmann and DeLong, 2003; Takakusaki, 2008). The primary cause of these diseases may not lay in the SNpr. However, understanding the basic principles of the basal ganglia function has allowed development of strategies, such as deep brain stimulation for modulation of the SNpr/GPi output and symptomatic treatment of the movement disorders (Bergman et al., 1990; Limousin and Foltynie, 2019). As the SNpr appears to contain neuron types with different or even opposite functions, access to the subtypes of the SNpr neurons may lead to more focused and effective therapies. As the SNpr gates many types of behaviors, it could even be targeted to treat symptoms of various neuropsychiatric disease including epilepsy, hyperactivity or even sleep disorders.

## Author contributions

JP and KA wrote the article and compiled the figure. JP conceptualized the study. Both authors contributed to the article and approved the submitted version.
